# Proximate Analysis of Five Wild Fruits of Mozambique

**DOI:** 10.1155/2013/601435

**Published:** 2013-07-25

**Authors:** Telma Magaia, Amália Uamusse, Ingegerd Sjöholm, Kerstin Skog

**Affiliations:** ^1^Department of Biological Science, Science Faculty, Eduardo Mondlane University, Praca 25 de Junho, P.O. Box 257, Maputo, Mozambique; ^2^Division of Applied Nutrition and Food Chemistry, Lund University, P.O. Box 124, 221 00 Lund, Sweden; ^3^Department of Chemistry, Science Faculty, Eduardo Mondlane University, Praca 25 de Junho, P.O. Box 257, Maputo, Mozambique; ^4^Division of Food Technology, Lund University, P.O. Box 124, 221 00 Lund, Sweden

## Abstract

Mozambique is rich in wild fruit trees, most of which produce fleshy fruits commonly consumed in rural communities, especially during dry seasons. However, information on their content of macronutrients is scarce. Five wild fruit species (*Adansonia digitata*, *Landolphia kirkii*, *Sclerocarya birrea*, *Salacia kraussii*, and *Vangueria infausta*) from different districts in Mozambique were selected for the study. The contents of dry matter, fat, protein, ash, sugars, pH, and titratable acidity were determined in the fruit pulps. Also kernels of *A. digitata* and *S. birrea* were included in the study. The protein content in the pulp was below 5 g/100 g of dry matter, but a daily intake of 100 g fresh wild fruits would provide up to 11% of the recommended daily intake for children from 4 to 8 years old. The sugar content varied between 2.3% and 14.4% fresh weight. The pH was below 3, except for *Salacia kraussii*, for which it was slightly below 7. Kernels of *A. digitata* contained, on average, 39.2% protein and 38.0% fat, and *S. birrea* kernels 32.6% protein and 60.7% fat. The collection of nutritional information may serve as a basis for increased consumption and utilization.

## 1. Introduction

In Mozambique, a large number of wild food plants are widely distributed throughout the country. The fruits and nuts are sold at informal markets during the harvest season and are consumed in various ways, and they are much appreciated by children [1–3]. The importance of wild fruits in the diet depends to a large extent on the availability of the fruits, since cultivated fruit trees are not particularly common in the dry regions of the country. Depending on the season, the fruits are eaten raw, pressed for juice, cooked with sugar, or used as flour to make porridge; the seeds or nuts are roasted to be eaten as snacks. The choice of fruit species varies according to region and cultural traditions [4]. 

Many wild fruits and nuts are good sources of carbohydrates, protein, fat, vitamins, and minerals that may be deficient in common diets [5]. There are some reports on the chemical composition of wild fruits from Southern African regions [5–9], but the literature data on the nutritional value of wild fruits in Mozambique is limited [4, 10]. People in many communities are not aware of the nutritional value of the fruits; for example, they often eat only the pulp of the fruits *Sclerocarya birrea *and* Adansonia digitata* while discarding the seeds, which contain a kernel with a higher protein and fat content than peanuts [1, 11].

The aims of this work were to perform a study on traditional utilization of wild fruits in Mozambique and to generate data on the proximate composition and other characteristics of five wild fruits as a basis for the selection of fruits suitable for processing. The long-term goal was to promote and increase the utilisation and consumption of indigenous fruits. The wild fruits selected for the present study were *Adansonia digitata, Landolphia kirkii, Salacia kraussii, Sclerocarya birrea, *and *Vangueria infausta*. These fruits are popular in Mozambique, and they play an important role in the diet, particularly in rural areas.

## 2. Materials and Methods

### 2.1. Species

Five wild fruit species were studied: *Adansonia digitata (A. digitata)* (family *Bombacaceae*, local name n'buyu or Malambe), *Landolphia kirkii (L. kirkii) *(family *Apocynaceae*, local name n'vhungwa), *Salacia kraussii (S. kraussii) *(family *Celastraceae*, local name n'phinsha), *Sclerocarya birrea (S. birrea)* (family *Anacardiaceae*, local name n'canhi), and *Vangueria infausta (V. infausta)* (family *Rubiaceae*, local name n'pfilwa). Ripe fruits were collected in 2008 and 2009, except for the fruits from *S. birrea*, which were collected only in 2009. *A. digitata* fruits, grown in the Tete district 1100 km from Maputo city, were bought at a local market in Maputo, and some fruits were collected in family orchards in the Vilankulos district, 700 km south of Maputo. *L. kirkii*, *S. kraussii,* and *V. infausta* fruits were collected in orchards in the Marracuene and Manhiça districts, 30 and 50 km south of Maputo. Fruits from *S. birrea *were obtained from a garden in Maputo city and *S. birrea *kernels, dried for 1–3 months, were obtained from a small family orchard in Manhiça. The fruits were collected in districts where there is an increased occurrence and consumption of them.

### 2.2. Sample Preparation

Unblemished fruits were selected and washed, the skin and seeds were removed, and the remaining parts were homogenized in a blender to obtain 100 g pulp of each type of fruit. Different numbers of fruit were used depending on fruit size and mass of pulp. The fruits from *A. digitata* had low moisture content and the pulp was ground into a fine powder and sieved (500 *μ*m meshes). The seeds from *A. digitata* and *S. birrea* were crushed and the kernels inside were removed, milled, and sieved (500 *μ*m meshes). Samples for determination of pH and titratable acidity were kept at room temperature and the analyses were performed on the day after collecting the fruit. The samples for the other analyses were vacuum-packed in plastic bags and stored at −18°C in a freezer. 

### 2.3. Analysis

To determine the dry matter content, 2 g samples were dried in an oven at 105°C until constant weight [12]. The samples were weighed before and after drying and the contents of dry matter were calculated. The protein content was determined in an Elementar Analyzer (Flash EA 1112 Series, Thermo Fisher Scientific, Sweden), by means of combustion of 25 mg samples. Aspartic acid (Thermo Fisher Scientific, Delft, The Netherlands) was used as a standard. The amount of protein was calculated by converting the amount of nitrogen by a factor 6.25. The fat content was determined gravimetrically after extracting 1 g samples with petroleum ether (Sigma-Aldrich Chemicals Co., St. Louis, MO, USA) at 40–60°C for 1 hour using a Soxhlet equipment (SoxtecTM 2055, Foss, Höganäs-Helsingborg, Sweden) [13]; rapeseed oil was used as a standard. The ash content was determined by combustion of 2 g samples in silica crucibles in a muffle furnace (Carbolite, Sheffield, England) for 24 hours at 550°C [12]. The pH was determined using a pH meter (Carison GLP 21, serial no. 147012, Barcelona, Spain). Titratable acidity, expressed in percentage of citric acid, was determined after titration of 10 g samples, dissolved in 100 mL water, with 0.1 M sodium hydroxide using phenolphthalein as indicator [12]. All determinations were performed at least in triplicate; the data are expressed as means ± standard deviations. Subsamples of fruits collected in 2009 were sent to an authorized laboratory for analysis of sugar content by high performance anion exchange chromatography (Dionex) with pulsed amperometric detection (HPAEC-PAD). The variation between duplicate determinations was below 15%. 

### 2.4. Statistical Analysis

Statistical analyses were performed using SPSS (version 13). Significant differences were evaluated with one-way analysis of variance (ANOVA) followed by Tukey's multiple comparisons test. A value *P* < 0.05 was considered to be significant. 

### 2.5. Interviews Regarding the Traditional Utilization of Wild Fruits

When the wild fruits were collected in the different districts, local people were interviewed about the traditional use of the fruits. For each fruit, 3–10 people were interviewed, both women and men of different ages, all of whom had grown up in rural areas. The aim of the interviews was to obtain information on knowledge concerning the occurrence of these wild fruit trees, their owners, and traditional habits regarding consumption, processing, and storage of wild fruits.

Examples of the interview questions are as follows. (i) What kind of wild fruits exist in this area? (ii) Who owns and takes care of these fruit trees? (iii) Who are the major consumers? (iv) How are the fruits eaten? (v) What is the typical amount harvested per day? (vi) How are the fruits stored? (vii) What kind of processing can be done? 

## 3. Results and Discussion

The results of the determinations of dry matter content and proximate composition (protein, fat, and ash) for pulp and kernels are summarised in Table 1. The values differed somewhat between the years, but the difference was not significant for any of the fruits. The dry matter content of *A. digitata* pulp was very high, on average 88.5%, while for the other fruits, it varied between 16.7% and 34.8%. The high dry matter content of *A. digitata* was in the same range as has been reported in studies in other countries, 85–95% [5, 7, 8, 14–18]. There is little literature data on the dry matter content of the other fruits, and for *S. birrea* our data agree with one report [7], while one report is showing a lower value [8]. For *V. infausta* our results are somewhat higher than reported in fruits from Malawi [5], Botswana [8], and Tanzania [17]. For *L. kirkii*, the dry matter content was comparable with the literature data [1]. The average dry matter content of *A. digitata* kernels was 92.0%, which is in agreement with other results, ranging from 85% to 97% [1, 7, 9, 14, 15, 18–21]. The average dry matter content of *S. birrea* kernels was 94.3%, which is at the same level as the results from other reports [1, 11, 15, 22, 23].

The protein content of the pulp was low in general (below 5%) for all the fruits. This is in agreement with results of other reports, [1, 5, 7, 15–18]. For *A. digitata*, however, there is a report showing a much higher protein content, 15.3% [24]. The protein content in the pulp was significantly lower than in the corresponding kernels (*P* < 0.05). *A. digitata* kernels contained on average 39.3% protein and *S. birrea* kernels 32.6%. For *A. digitata*, our results are in accordance with other results [7, 18], but there are also reports showing lower protein content, 13–27% [14, 15, 19, 21], and one report showing higher protein content, 48.3% [20]. The protein content in the kernels of *S. birrea* was at the same level as found in another report [23]. The high protein content in the kernels is at the same levels as reported for soya beans, around 33% [11], which means that the kernels may be a potential source of protein and can be used to improve the diet in rural communities. For example, a daily intake of 100 g of fresh pulp from the wild fruits studied here would provide around 2–11% of the recommended daily intake (RDI) for children from 4 to 8 years old, while 20 g of *A. digitata* or *S. birrea* kernels would provide 32–39% of the RDI for children of the same age [25]. The protein quality of the kernels seems to be good since high amounts of the essential amino acid lysine as well as of arginine, glutamic acid, and aspartic acid have been reported for *A. digitata* kernels [15]. *S. birrea* kernels have been shown to contain high amounts of the essential amino acids phenylalanine, lysine, and threonine [3, 6].

The fat content in the pulp was below 2% for all the fruits. The literature data on *A. digitata* and *S. birrea* pulp generally show fat contents below 1% [1, 7, 14–16, 18], while some reports show a higher fat content in *A. digitata*, 4% [5, 17, 24]. The pulp of wild fruits is typically low in fat and protein [5], while the kernels are good sources of fat and protein [10]. In the present study, the average fat content was 38.0% in kernels of *A. digitata* and 60.7% in kernels of *S. birrea*, and the fat content in the kernels was significantly higher than that in the pulp (*P* < 0.05). Our data on kernels are in agreement with results from other studies [1, 3, 22, 23], while the fat content was lower in two reports [20, 21] and higher in one [18]. The fat quality of the kernels is good according to the literature data: *A. digitata* kernels are rich in palmitic, oleic, and linoleic acid (essential fatty acid) [15], and *S. birrea* kernels are rich in oleic and linoleic acid [3]. 

The average ash content ranged from 3.0% to 7.8%. For *A. digitata* pulp and kernel, the results are at the same level as in other reports [7, 8, 14–17, 21]. The ash content was somewhat lower than that in some other reports for *S. birrea* [7, 8, 23] and *V. infausta* pulp [5]. The high ash content indicates that the fruits and kernels may be good sources of minerals.

The pH of the pulps showed an acidic character (around pH 3) except for *S. kraussii*, for which the pH was slightly above 6. The acidic character is in accordance with data on pulps from *A. digitata, S. birrea, *and* V. infausta* [8]. The pH of fruits generally varies between 2.5 and 4.5 due to their content of organic acids [26]; the low pH enhances the microbiological and physicochemical stability [27]. 

The titratable acidity of the pulp, which contributes to the acidity of the aroma, ranged from 0.6% to 1.7%. In another report, also using citric acid, the titratable acidity was 7.8% for *A. digitata, *0.9% for *S. birrea,* and 1.7% for *V. infausta* [8]. Comparable data were 0.3% for mango pulp [28] and 0. 7% for orange juice [29].

Table 2 shows the sugar content of the investigated fruits expressed as g sugar/100 g pulp. The highest total sugar content was found in *A. digitata* and *L. kirkii,* 10.3 and 14.4 g/100 g, respectively. The value for *A. digitata* is much lower than that reported in another study, where the total sugar content was around 30% [16]. The highest sucrose content, 4.3 g/100 g, was found in *A. digitata*, while for the other fruits it was lower than 3 g/100 g. As expected, only very low amounts of maltose and lactose, below 0.04 g/100 g, were detected; the most abundant sugars in fruits, are glucose, fructose, and sucrose in various proportions, depending on species [30]. 

The sugar content, data on pH, and titratable acidity are essential characteristics, indicating the possibility for future use of these wild fruits. The sugar content is important for the development of the aroma and taste, and in product development it is important to find a good balance between pH, sugars, and titratable acidity to receive an optimal taste. The wild fruits in our study have different profiles regarding these characteristics but are in accordance with the literature data on some traditional fruits for juice production, for example, papaya, mango, pineapple, and orange [29–32]. 

*Interviews.* The interviews revealed that the majority of the fruits came from the forest and that wild fruits provide food for everyone, especially for children because they are more free to go into the forest to collect fruit. Some people said that in periods of hunger, the leaves, fruits, and seeds are used as food as well as for medical applications. Wild fruits can serve as a source of income for many families, depending on the amount of fruit that each family can reap in the forest or in the area surrounding their house. The fruit is usually sold early in the morning at informal markets to which people come to buy fruit to sell in the markets in the cities.

Different fruits are used in different ways; see Table 3. *A. digitata* fruits are sold in different forms, for example, whole fruit, pulp with seeds embedded, and fine powder made from the pulp packed in plastic bags. The seed-containing pulp is often consumed fresh and soaked in warm water to remove the seeds, and the remaining “milk” can be mixed with sugar to form juice, or boiled with maize flour or sorghum to make a porridge given to children before they go to school. Another way of using the pulp is to dilute it with warm water to prepare a juice, which is filtered, mixed with sugar and packed in small plastic bags and frozen. This sweet ice is commonly sold in informal markets, and it is served as refreshment consumed by both children and adults. 

The seeds are crushed and the kernels inside can be consumed fresh or roasted. They can be milled to powder and mixed with a small amount of water and boiled with local plant food to make a sauce consumed with boiled maize flour. The seeds are also boiled with a small amount of water or milk to make porridge for children.

People in rural communities usually eat *L. kirkii* fruits fresh, but when large amounts of these fruits are available, they are squeezed and fermented to produce a local alcoholic drink that is consumed at social gatherings. Rural people of all ages eat *L. kirkii* fruit while walking to and working on their grassland and cattle farm plots, which are sometimes far away from their homes.

The fruits from the small *S. kraussii* bushes are more easily accessible to children. Many school children eat the fresh fruits on their way to and from school or while grazing cattle. 

Fresh fruit from *S. birrea* is squeezed to make juice or fermented to produce a popular alcoholic drink. After juice extraction and fermentation, the juice may be stored in sealed clay pots or plastic containers for up to a year. The kernels can be eaten fresh or roasted or ground in a mortar together with water, boiled with local plant food to make a sauce. The kernels can also be used to produce oil for cooking.

In the southern part of Mozambique, *V. infausta* fruits are commonly consumed fresh, as juice, but they are also often fermented to produce alcoholic drinks. Fresh fruit is soaked in water, and the skin and seeds discarded before the preparation of a juice which is mixed with water and sugar or milk and served as porridge for children. Excess fruit is dried and stored for later use.

## 4. Conclusion

The wild fruits studied are consumed by people living in different districts of Mozambique and form a part of their normal diet. Our data on the proximate composition, pH, titratable acidity, and sugar content are consistent with the few reports available in the literature. We observed low but not significant variations between the growth locations and the harvest year, and these variations may be due to differences in climate, soil, and weather conditions. The findings of this study have shown that the analyzed fruits, and especially the kernels, are good sources of protein and fat. In Mozambique malnutrition is responsible for one-third of deaths in children under five years, and based on the above results, it may be concluded that promotion of consumption and processing of these fruits, to various products, may help to improve the diet and alleviate nutrient deficiencies. 

## Figures and Tables

**Table 1:** Dry matter content, protein, fat, and ash expressed in g/100 g dry matter (*n* = 3).

Sample Location_year	Dry matter (%)	Protein	Fat	Ash
*Adansonia digitata *pulp				
Tete_2008	89.8 ± 0.1	2.4 ± 0.0	0.5 ± 0.1	5.5 ± 0.1
Tete_2009	89.1 ± 0.0	2.2 ± 0.1	0.7 ± 0.6	7.4 ± 0.0
Vilanculos_2009	86.5 ± 0.1	2.1 ± 0.0	0.5 ± 0.1	7.4 ± 0.0
*Landolphia kirkii *pulp				
Marracuene_2008	27.7 ± 0.2	2.1 ± 0.2	0.9 ± 0.1	2.9 ± 0.0
Marracuene_2009	23.9 ± 0.0	NA	0.4 ± 0.2	3.5 ± 0.0
Manhiça_2009	20.1 ± 0.3	1.7 ± 0.0	1.3 ± 0.1	3.0 ± 0.0
*Salacia kraussii *pulp				
Marracuene_2008	16.4 ± 0.3	3.7 ± 0.0	0.8 ± 0.5	4.8 ± 0.2
Manhiça_2008	17.3 ± 0.1	2.3 ± 0.0	1.5 ± 0.6	3.4 ± 0.0
Manhiça_2009	16.5 ± 0.1	2.1 ± 0.1	0.7 ± 0.2	3.6 ± 0.0
*Sclerocarya birrea *pulp				
Manhiça_2009	16.8 ± 0.1	1.4 ± 0.1	0.9 ± 0.3	3.0 ± 0.1
*Vangueria infausta *pulp				
Marracuene_2008	37.4 ± 0.5	2.9 ± 0.2	0.5 ± 0.8	7.8 ± 0.6
Marracuene_2009	30.0 ± 0.1	2.2 ± 0.3	0.7 ± 0.1	5.3 ± 0.0
Manhiça_2008	37.3 ± 0.9	4.7 ± 0.4	0.7 ± 0.2	5.7 ± 0.4
Manhiça_2009	34.5 ± 0.7	3.3 ± 0.8	0.2 ± 0.0	3.2 ± 0.2
*Adansonia digitata *kernel				
Tete_2008	93.6 ± 0.0	36.7 ± 0.9	35.0 ± 0.2	7.7 ± 0.0
Tete_2009	91.7 ± 0.1	38.6 ± 0.8	39.9 ± 6.2	7.2 ± 0.0
Vilanculos_2009	90.6 ± 0.0	42.7 ± 0.7	39.0 ± 7.0	8.5 ± 0.2
*Sclerocarya birrea *kernel				
Manhiça_2008	93.6 ± 0.2	30.1 ± 0.2	58.3 ± 1.3	3.8 ± 0.0
Manhiça_2009	95.0 ± 0.0	35.0 ± 0.1	63.1 ± 0.1	3.5 ± 0.0

**Table 2:** Sugar content of selected fruits obtained in 2009 from Manhiça expressed in g sugar/100 g pulp (*n* = 2).

Sample	Dry matter (%)	Glucose	Fructose	Sucrose
*Adansonia digitata *	88.0	3.0	3.0	4.3
*Landolphia kirkii *	18.0	7.5	5.7	1.2
*Salacia kraussii *	9.0	3.7	3.9	<0.1
*Sclerocarya birrea *	8.0	0.5	0.4	1.4
*Vangueria infausta *	31.0	1.4	1.4	2.7

The variation between duplicate determinations was below 15%.

**Table 3:** Traditional consumption and use of the studied fruits.

Species	Utilisation
*Adansonia digitata *	Pulp: fresh, diluted, and sweetened to juice, frozen to sweet ice, cooked to porridge, and fermented to an alcoholic drink
	Kernels: fresh or roasted, milled and boiled to sauce or porridge

*Landolphia kirkii *	Fresh or fermented to an alcoholic drink

*Salacia kraussii *	Eaten fresh

*Sclerocarya birrea *	Pulp: fresh, squeezed to juice, and fermented to an alcoholic drink
	Kernels: fresh or roasted, cooking oil can be extracted

*Vangueria infausta *	Fresh, soaked, squeezed to juice, mixed with sugar, water, or milk to a porridge, and fermented to an alcoholic drink
